# HS−SPME/GC−MS Reveals the Season Effects on Volatile Compounds of Green Tea in High−Latitude Region

**DOI:** 10.3390/foods11193016

**Published:** 2022-09-28

**Authors:** Jie Wang, Xiaohan Li, Ying Wu, Fengfeng Qu, Lei Liu, Baoyi Wang, Peiqiang Wang, Xinfu Zhang

**Affiliations:** 1 College of Horticulture, Qingdao Agricultural University, Qingdao 266109, China; 2 College of Agriculture, Tennessee State University, Nashville, TN 37209, USA; 3 Bureau of Agriculture and Rural Affairs of Laoshan District, Qingdao 266061, China

**Keywords:** green tea, season, volatile compounds, gas chromatography–mass spectrometry (GC−MS)

## Abstract

This study investigates the volatile compounds of green tea produced with different leaves from spring, summer, and autumn in high−latitude region. A total of 95 volatile compounds were identified by gas chromatography–mass spectrometry (GC–MS). Spring, summer and autumn green tea contained 68, 72 and 82 volatile compounds, respectively. Principal component analysis (PCA), partial least squares−discrimination analysis (PLS−DA), and hierarchical cluster analysis (HCA) classified the samples and showed the difference. And 32 key characteristic components were screened out based on variable importance in the projection (VIP) values higher than 1.0. The characteristic volatile compounds of spring green tea including 18 components, such as geranylacetone, phenethyl alcohol, geraniol, β−ionone, jasmone, 1−octen−3−ol and longifolene. 13 components such as 2−methylfuran, indole, 1−octanol, D−limonene and ethanethiol were the key compounds in summer green tea. And 2,4,6−trimethylstyrene was the major differential volatile compounds in autumn green tea. The results increase our knowledge of green tea in different seasons and provide a theoretical basis for production control of green tea.

## 1. Introduction

Green tea, classified as unfermented tea, accounts for 60% of China’s annual tea production, and is widely appreciated by consumers owing to its unique aroma [1,2]. Aroma is a key indicator in determining the sensory quality and economic value of the tea [3]. Tea aroma is basically related to volatile compounds. Although the volatile compounds approximately represent only 0.01% of dry weight of tea, they play an important role in the quality of tea due to their low threshold values [4,5,6,7]. At present, almost 700 volatile compounds have been detected in tea but only about 300 in green tea [8,9]. These volatile compounds can be divided into the following classes: alcohols, aldehydes, ketones, esters, hydrocarbons, sulfur compounds, and nitrogen compounds [2,7]. Due to the complexity of aroma components and the differences in aroma of different teas, identification of aroma has attracted great interest [10].

Previous studies have examined volatile compounds in green tea, and components such as linalool nonanal, (Z)−3−hexenyl hexanoate, β−ionone, geraniol, and cis−jasmone were identified as major volatiles [11,12]. Chinese green tea emits a variety of aroma types such as chestnut−like, clean, floral aroma, which are attributed to their different volatile profiles [13,14]. Some studies have reported that ethylbenzene, linalool, trans−β−ionone, dimethyl sulfide, heptanal, benzaldehyde, 2−pentylfuran, 2−ethylfuran, and (E,E)−3,5−octadien−2−one are the key odorants responsible for the chestnut−like aroma [13,15]. It has been shown that linalool, nonaldehyde, 1−octen−3−ol, D−limonene, methyl salicylate contribute to the clean aroma [16,17]. On the other hand, tea aroma is influenced by many factors. In recent years, extensive research has focused on the effects of different processing methods, origins, and cultivars on the volatiles of green tea [18,19,20]. However, there are few studied on the effect of season on the aroma of green tea.

The sensory quality (aroma and taste) of tea is greatly affected by the climatic changes of different seasons [21,22], and the production season of tea is an important concern for consumers when purchasing tea [6]. According to the harvest seasons of fresh tea leaves, tea can be divided into spring tea, summer tea, and autumn tea. Summer tea and autumn tea are more bitter and astringent than spring tea, which results in a decrease in their economic value [23,24,25]. Generally, spring tea exhibits a sweet and floral aroma, while summer tea exhibits hay and grassy notes [26]. Different ecological environments such as climate, humidity, moisture, and temperature in different seasons lead to different enzyme characteristics and chemical composition of tea trees [21,27]. This ultimately results in the difference of tea aroma compositions and contents. However, there is no systematic study on the aroma of green tea in different seasons, and the relationship between the harvest season and volatile compounds of green tea remains unclear.

Shandong Province is located at the north latitude of 34–38°, belonging to the high latitude tea area. Due to its unique climate and geological conditions, with large temperature difference between day and night, Shandong green tea possesses an excellent aroma quality. Tea plants grown in the Shandong area are plucked late because of the low temperature in early spring, but the harvesting period is long. The quality characteristics of green tea produced in the three seasons of spring, summer and autumn are different and have their own distinctive aroma profile. However, there has been no systematic evaluation of the characteristic volatile compounds of Shandong green tea. Hence, the aroma of green tea from different seasons in the high−latitude region needs to be elucidated.

Multivariate statistical analysis methods such as principal component analysis (PCA) and partial least squares−discrimination analysis (PLS−DA) have been proven to be efficient and rapid methods to classify food samples and highlight their differences [28,29,30,31]. PCA is an unsupervised classification model that highlights similarities and differences between and within samples [14,32]. PLS−DA as a supervised model maximizes the differences between groups and screens the key compounds responsible for separation [33]. The variable importance in the projection (VIP) of PLS–DA can quantify the contribution of each variable to the classification. As the volatile compounds of tea are numerous and complex, PCA and PLS−DA are valid means to investigate the difference among tea samples [34,35].

In this study, headspace solid phase microextraction (HS−SPME) combined with gas chromatography−mass spectrometry (GC−MS) were employed to investigate the characteristic differences between the green teas produced in different seasons. These methods have been widely applied in the identification of volatile compounds in foods with high accuracy and efficiency [36,37]. Our results may provide a theoretical basis for quality control and flavor improvement of green tea produced in high latitudes from different seasons.

## 2. Materials and Methods

### 2.1. Samples and Tea Preparation

The tea variety Longjing−changye, one of the main varieties cultivated in the high latitude tea area, was selected as the tea source for the experiment. The fresh tea leaves (one bud and two leaves) were collected from Laoshan area (Qingdao City, Shandong Province, China) in May, July, and September of 2018, respectively. All samples were prepared using identical processing techniques. First, fresh leaves were withered for 3 h under natural conditions, and then the withered leaves were fixed at approximately 220–240 °C. Fixed leaves were rolled lightly for 15 min, then rolled heavily for 30 min, finally rolled lightly for 20 min. After rolling, each tea leaf was toasted using a drum at 110 °C for 30 min, finally green tea was obtained by redrying at 90 °C for 1 h. All tea samples were stored at −80 °C until analyzed.

### 2.2. Sensory Evaluation

According to the China Sensory Review Standards (GB−T/23776−2018), all samples were evaluated by five experienced experts (three males and two females) who were trained and authenticated by professional organizations [38,39]. The sensory evaluation was carried out in a professional and quiet panel room with a temperature of 20–25 °C. First, 200 g of tea sample was evenly weighed to evaluate the appearance. 3.0 g of tea sample was infused with 150 mL of boiled water for 4 min, the tea soup was filtered out at the same speed, and the infused leaves was left in the tea pot. Tea samples were blind−coded with random numbers. The appearance, liquor color, aroma, taste, and infused leaves qualities of the green teas were evaluated by experts. The total sensory score was evaluated by quality scores using a 100−point scale, in which 10% accounted for the appearance of the dry tea, 30% for the aroma, 15% for the liquor color, 35% for the taste and 10% for the infused leaves. Samples were assessed three times through blind evaluation.

### 2.3. HS−SPME Method

The extraction of green tea volatile compounds was conducted by reported HS−SPME method with minor modifications [40,41]. Briefly, 6.0 g of the ground tea sample were placed in a 100 mL vial. After adding 4 g NaCl and 20 mL 100 °C distilled water, the rotor of the magnetic stirrer was put into it, then the vial was put in a water bath at 60 °C for 5 min, followed by exposure to a 75 μm CAR/PDMS coating fiber for 1 h. After the extraction was completed, the SPME fiber was inserted into the injector of the gas chromatograph at 250 °C for 5 min to desorb the analytes. Each sample was repeated three times. 

### 2.4. GC–MS Analysis

The GC−MS analytical procedure was based on previous study [40]. Chromatographic column is Agilent DB−5MS capillary column (30 m × 0.25 mm × 0.25 μm). The temperature of GC injector was 250 °C. Helium (percentage purity > 99.999%) was used as carrier gas at a constant flow rate of 1 mL/min. The oven temperature was held at 50 °C for 5 min, increased to 180 °C at 3 °C/min (held for 2 min), then increased to 250 °C at 10 °C/min (held for 3 min), and finally increased 280 °C at 10 °C/min (held for 3 min). The mass spectrometer was operated in an electron−impact mode of 70 eV. The temperatures of the ion source, quadrupole, and interface were 230 °C, 150 °C, and 280 °C, respectively, and the acquisition mode was full scan (from 30 to 400 aum). 

### 2.5. Data Processing

The raw data acquired by GC–MS were first deconvolved using Agilent Mass Hunter Qualitative Analysis software (Agilent Technologies Inc. Palo Alto, CA, USA). Volatile compounds were tentatively identified by comparing their mass spectra (MS) and the practical retention indices (RI, determined by n−Alkanes C_6_−C_25_) with information from National Institute of Standards and Technology (NIST) library. RI was calculated with the retention time of each compound according to previous literature [42]. Relative contents of the identified compounds were obtained by dividing the area of a single peak by the total areas.

All identified compounds were used for statistical analysis. One−way ANOVA (Duncan’s multiple range tests) was used for data analysis by SPSS 25.0 software (Demo version, Armonk, NY, USA). *p* < 0.05 was considered to be significantly different. According to the composition and relative content of volatiles, PCA and PLS−DA models were conducted by SIMCA−P 14.1 software (Umetrics, Umea, Sweden). The key volatile compounds responsible for each sample were screened by variable influences in projection (VIP) > 1.0 [38]. A hierarchical cluster analysis (HCA) heat map was generated using Multi Experiment Viewer (MEV) software (Oracle Corporation, Redwood Shores, CA, USA).

## 3. Results

### 3.1. Sensory Quality Analysis

The sensory evaluation results of green teas are shown in Table 1. The appearance, liquor color, aroma, taste, infused leaves of green tea in different seasons have significant differences. Spring tea had the highest total score followed by autumn tea and summer tea. Interestingly, green teas produced in different seasons had different aroma characteristics. Spring tea had an obvious chestnut−like aroma and scored the highest for aroma, while summer tea and autumn tea had clean and floral aromas, respectively, both of which performed moderately. A similar trend was also observed in the score of liquor color and taste, which followed the order of spring tea > autumn tea > summer tea. The liquor color of spring tea, summer tea and autumn tea had the tender yellowish, blue dull, yellowish green, respectively. As for taste, spring tea was fresh, thick and had a sweet aftertaste; summer tea was astringent and strong; autumn tea had a bitter aftertaste and was not strong enough. In addition, the score order of appearance and infused leaves was spring tea > summer tea > autumn tea. The appearance of spring tea was tight, thin and tender green, while summer tea and autumn tea were black green and coarse, respectively. The infused leaves of spring tea, summer tea and autumn tea were tender green, yellowish green, and dull green, respectively. The results showed that different picking seasons are important factors affecting the quality of tea, not only the score of tea aroma was different, especially the type of aroma was changed.

### 3.2. Analysis of Volatile Compounds in Green Teas Produced in Different Seasons

The volatile components of all samples were detected by HS−SPME/GC−MS. A total of 95 compounds were tentatively identified, including 40 hydrocarbons, 17 alcohols, 10 esters, 8 aldehydes, 5 ketones, 4 phenols, 2 sulfur compounds, and 9 other compounds in three green teas (Table 2).

**Figure 1:**
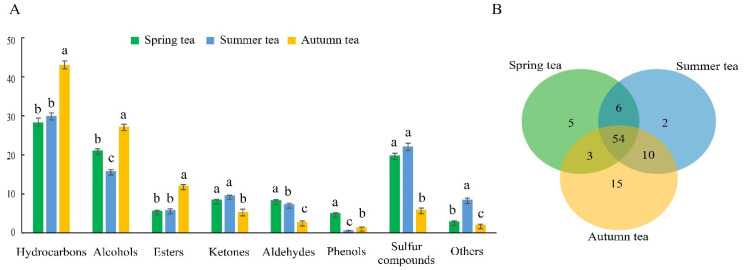
Comparison of the volatile components in tea samples from different seasons: (**A**) the classification of volatile components; and (**B**) Venn diagram of volatile components. Different lowercase letters in the same volatile categories indicate significant differences at *p* < 0.05 level.

Figure 1A showed that the relative contents of volatiles were significantly different in three green teas. Of the 95 volatiles, hydrocarbons were present with the largest proportion and ranged from 28.35% to 43.04%, being lowest in spring tea and highest in autumn tea. Alcohols were the second most abundant class of compounds in green teas, and the relative content was in the order of autumn tea (27.21%) > summer tea (21.12%) > spring tea (15.85%). As for aldehydes, the content in spring tea (8.41%) was the highest, followed by summer tea (7.37%) and autumn tea (2.81%). A total of 10 esters were detected in this study, which had higher content in autumn tea (12.14%), and there was little difference between the spring tea (5.69%) and summer tea (5.87%). Ketones occupied a little proportion in green tea. Summer tea had the highest ketones (8.53%), while autumn tea had the lowest ketones (5.41%). Additionally, the sulfur compounds of spring tea (18.95%) were obviously higher than that of autumn tea (5.91%). As for the rest of the identified compounds, the content of phenols (0.67–5.11%), and other (2.06–8.57%) volatile substances were low.

A Venn diagram was performed to visualize the distribution of compounds in green tea during the three seasons (Figure 1B). The number of volatile compounds in spring tea, summer tea, and autumn tea was 68, 72, and 82 compounds, respectively. There were 54 common volatile compounds in three green teas. Interestingly, spring tea and autumn tea had 3 common compounds, spring tea and summer tea had 6 common compounds, and summer tea and autumn tea had 10 common compounds. It is worth noting that 5, 2 and 15 compounds were detected only in spring tea, summer tea, and autumn tea, respectively. 2−Phenylethyl bromoacetate, isobutyl (m−tolyl) sulfide, nonanoic acid, 4−amino−2−methylphenol, and cis−5−ethenyltetrahydro−α, α−5−trimethyl−2−furanmethanol were existed only in spring tea (Table 2). 1−octanol, m−Anisidine were only detected in summer tea (Table 2). Cyclooctane, neroloxide, α−murulene, methyl mandelate, 3−ethenyl−1,2−dimethyl−1,4−cyclohexadiene, 1−methyl−1−cyclohexene, 2−methyl−cyclopentanol, 3−ethenyl−1,2−dimethyl−1,4−cyclohexadiene, α−calacorene, α−muurolene, 3,4−dimethyl−phenol,α−ylangene, 2,6,6−Trimethyl−1−cyclohexene−1−acetaldehyde, benzyl cyanide, and 2,4−dimethyl−1−(1−methylethenyl)−cyclohexene were the exclusive components to autumn tea (Table 2). All of this indicated that there are significant differences in the categories and contents of green tea aroma in three seasons.

### 3.3. Multivariate Statistical Analysis

#### 3.3.1. Principal Component Analysis

**Figure 2:**
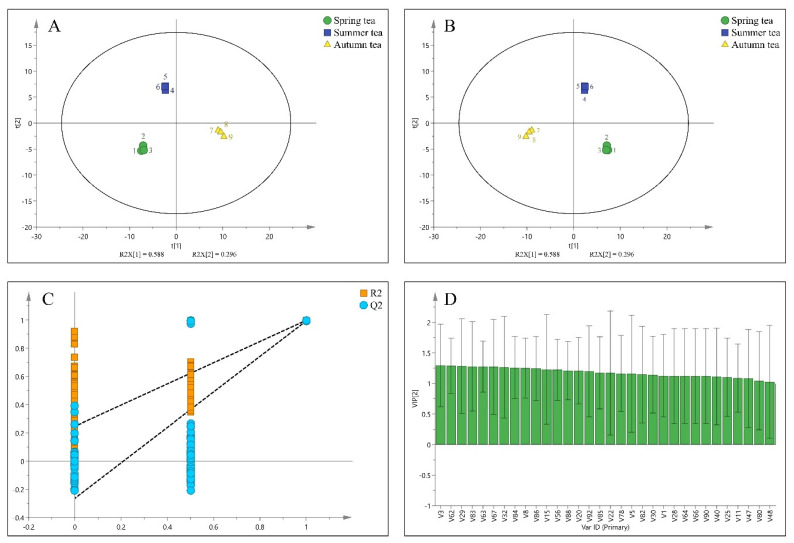
Multivariate analysis of spring, summer, and autumn tea samples: (**A**) PCA score plot; (**B**) PLS−DA score plot, R^2^X = 0.844, R^2^Y = 0.995, Q^2^ = 0.991; (**C**) cross−validation plot of PLS−DA model with 200 times of calculations by using permutation test (R^2^ = 0.276, Q^2^ = −0.259); and (**D**) the variable important in the projection (VIP > 1) key volatile components (numbers in Figure 2D are serial numbers of the aroma components in Table 2).

The PCA model was established based on the relative content of volatile components. As shown in Figure 2A, tea samples of different seasons were successfully divided into three groups, indicating that each group possessed a unique aroma profile. Spring, summer, and autumn tea was in the third, second and fourth quadrants, respectively. Principal component 1 (PC1) and principal component 2 (PC2) explained 58.8% and 29.6% of the total variation (88.4%), respectively. With the passage of seasons, samples of different seasons were distributed from left to right on PC1.

#### 3.3.2. Partial Least Squares−Discrimination Analysis

Partial least squares−discrimination analysis (PLS−DA) was adopted to compare the volatile profiles of green tea in three seasons. In Figure 2B, the score plot showed that green tea samples in three seasons were completely separated. And the model parameters (R^2^Y = 0.995, Q^2^ = 0.991) indicated the robustness of the model. Then, the effect of modeling was evaluated by the method of substitution test. The low intercepts (R^2^ = 0.276, Q^2^ = −0.259) was obtained through 200 times cross−validations, which demonstrated that there was no overfitting phenomenon, and this model was reliable (Figure 2C).

Variable importance in the projection (VIP) can quantify the contribution of each variable of PLS−DA to classification. It is generally considered that the variable with VIP value greater than 1.0 plays a key role in classification [37,43]. In this study, 32 components with VIP values > 1.0 were identified based on the established PLS−DA model (Figure 2D). These 32 key differential volatile compounds played a crucial role in the formation of aroma quality of green tea in different seasons. Among them, 5−methylthiazole, 2−methyl−furan, m−Anisidine, 2,6−dimethyl−6−(4−methyl−3−pentenyl)−bicyclo [3.1.1.]hept−2−ene, geraniol, indole, 3−methyl−1−butanol, 1−octanol, geranylacetone, and β−Ionone were the major differential compounds among three green teas.

#### 3.3.3. Hierarchical Clustering Analysis

**Figure 3:**
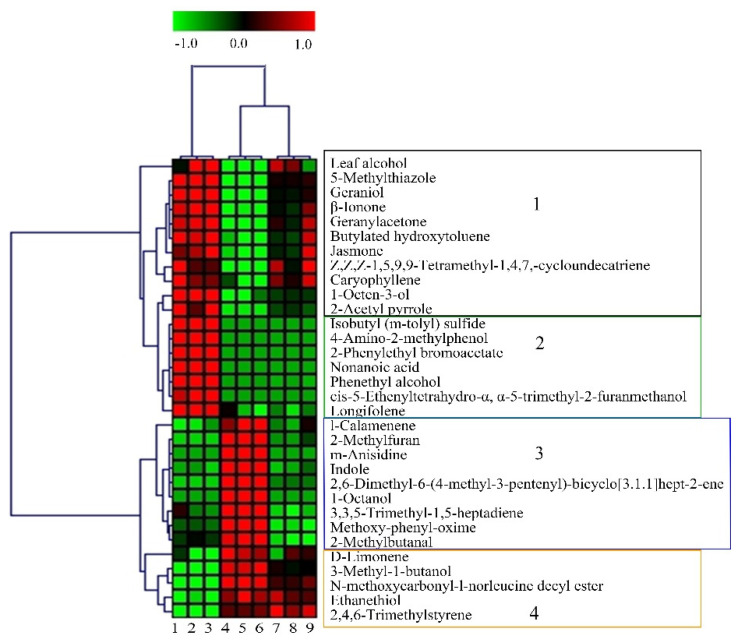
HCA analysis of 32 key compounds of green tea produced in different seasons (1–3 are spring tea, 4–6 are summer tea, 7–9 are autumn tea).

Hierarchical cluster analysis revealed the distribution of 32 key differential compounds among spring tea, summer tea and autumn tea. In Figure 3, 32 key differential compounds were clearly divided into four groups. Group 1 consisted of leaf alcohol, 5−methylthiazole, geraniol, β−ionone, geranylacetone, Z,Z,Z−1,5,9,9−Tetramethyl−1,4,7,−cycloundecatriene, jasmone, caryophyllene, 2−acetyl pyrrole, butylated hydroxytoluene, 1−octen−3−ol, which were mainly alcohols, hydrocarbons, and ketones. The order of contents of these compounds was spring tea > autumn tea > summer tea. Group 2 included longifolene, cis−5−ethenyltetrahydro−α, α−5−trimethyl−2−furanmethanol, phenethyl alcohol, isobutyl (m−tolyl) sulfide, nonanoic acid, 4−amino−2−methylphenol, 2−phenylethyl bromoacetate. These compounds had higher contents in spring tea than in summer tea and autumn tea. Group 3 contained N−methoxycarbonyl−l−norleucine decyl ester, 2−methylfuran, m−anisidine, 2,6−dimethyl−6−(4−methyl−3−pentenyl)−bicyclo [3.1.1]hept−2−ene, indole, 1−octanol, l−calamenene, 3,3,5−trimethyl−1,5−heptadiene, methoxy−phenyl−oxime. These compounds had higher contents in summer tea than in summer tea and autumn tea. Group 4 composed of 2,4,6−trimethylstyrene, 3−methyl−1−butanol, ethanethiol, D−limonene, N−methoxycarbonyl−l−norleucine decyl ester. These compounds had higher contents in summer tea and autumn tea than in spring tea. In conclusion, 18 volatiles including geranylacetone, phenethyl alcohol, geraniol, β−ionone, jasmone, 1−octen−3−ol, longifolene were the key compounds in spring tea; 13 volatiles including 2−methylfuran, indole, 1−octanol, D−limonene, ethanethiol were key compounds in summer tea; the key compound of autumn tea was 2,4,6−trimethylstyrene.

## 4. Discussion

Differences in the content and composition of volatile compounds result in different types of tea aroma. In our study, 32 key compounds were identified based on multivariate statistical analysis. Geraniol (Sweet), β−ionone (woody, violet−like), jasmone (woody, floral), and 1−octen−3−ol (mushroom−like, earthy) had higher content in spring tea, which might be key source of the chestnut−like characteristic of spring tea [33,44,45]. Previous study demonstrated that geranylacetone, phenethyl alcohol, 1−octen−3−ol, and longifolene were the key odorants of the chestnut−like aroma [13,15,38,46], which was consistent with our findings. Additionally, the key compounds of summer tea including 1−octanol (green), D−limonene (citrus−like, fresh), 2−methylfuran (chocolate), ethanethiol (sulfurous, fruity) play an important role in the aroma profile of summer tea. And D−limonene has been reported to contribute to the clean aroma of green tea [38], which was in keeping with our results. The research showed that (Z)−methyl epijasmonate was responsible for the orchid aroma of green tea [47]. In present study, 2,4,6−trimethylstyrene was the key compounds of autumn tea. The difference in key component from those previously reported for floral aroma may be due to differences in tea cultivars and origins.

The aroma quality is affected by the harvest season, cultivar, origin, manufacturing process [48,49], of these, season is a crucial factor. In tea leaves, aroma components are mainly produced through enzyme−assisted transformation and degradation of precursors [21]. Glycosides, carotenoids, amino acids, fatty acids, and terpene derivatives are the main tea aroma precursors [50]. The synthesis of these aroma precursors is affected by seasonal climate changes such as light, temperature and humidity, which further affect the generation of volatiles. The concentration of glycoside precursors and glycosidic enzymes seasonally change in tea leaves, expressed from high to low as spring > summer > autumn [50,51]. In our study, the contents of linalool, geraniol, benzyl alcohol, and phenethyl alcohol synthesized from their corresponding glycoside precursors showed the similar trend [51,52]. β−ionone is an important contributor to the aroma of green tea due to its low odour threshold [53], which comes from the primary oxidation of β−carotene [50]. In previous studies, carotenoids are regulated by light and temperature, and had highest content in spring tea [54,55,56]. This was similar to our results that the content of β−ionone was most abundant in spring tea. Additionally, the aroma score of summer tea was the lowest in this study. Amino acids are important substances for the formation of tea aroma though the Maillard reaction [57]. However, studies have shown that strong light in summer results in less amino acids in summer tea [19,58], reducing the source of aroma in summer tea, which is consistent with our study. At present, the exact seasonal climate effects on volatile compounds in tea have not been reported, the biosynthesis pathways of key aroma components in different seasons needs further study to clarify.

## 5. Conclusions

Season is an important factor affecting the aroma of tea. In this study, according to the sensory evaluation results, spring tea, summer tea, and autumn tea showed chestnut−like, clean, and floral aroma, respectively, and the aroma score was ranked as spring tea > summer tea > autumn tea. 32 key compounds were identified. Among them, 18 volatile compounds including geranylacetone, phenethyl alcohol, geraniol, β−ionone, jasmone, 1−octen−3−ol, longifolene were the key compounds in spring tea; 13 volatile compounds including 2−methylfuran, indole, 1−octanol, D−limonene, ethanethiol were key compounds in summer tea; the key component of autumn tea was 2,4,6−trimethylstyrene. This study enriched the aroma theory of green tea from the high latitude region and provided scientific basis for quality control of green tea production.

## Figures and Tables

**Table 1:** Sensory evaluation of green teas produced from different seasons.

Name	Appearance		Liquor Color		Aroma		Taste		Infused Leaves		Total Score
	Remarks	Score	Remarks	Score	Remarks	Score	Remarks	Score	Remarks	Score	
Spring tea	Tight, thin, tender green	92.00 ± 0.71a	Tender yellowish	89.80 ± 0.84a	Chestnut−like, tender	92.80 ± 0.84a	Fresh, thick, sweet aftertaste	91.80 ± 0.84a	Tender green	91.80 ± 0.84a	91.84a
Summer tea	Tight, thin, black green	89.60 ± 1.14b	Blue dull	86.40 ± 0.89bc	Clean, slight harsh odour	87.20 ± 0.84c	Astringent, strong	85.40 ± 1.14c	Yellowish green, little blueish	89.20 ± 1.06b	87.38c
Autumn tea	Coarse, loose, yellowish green	87.00 ± 1.00c	Yellowish green, slight blueish	88.40 ± 1.14b	Floral, little green odour	90.20 ± 1.48b	Bitter aftertaste, not strong enough	88.20 ± 0.84b	Dull green	87.20 ± 0.84c	88.32b

Note: Data are presented as mean value ± standard deviation (mean ± SD). Different small letters indicate significant differences (*p* < 0.05)

**Table 2:** Volatile components and their relative contents of tea in different seasons.

No.	Retention Time	RI ^1^	Compounds ^2^	ID ^3^	Relative Content (%) ^4^			VIP	Odor Description ^5^
					Spring Tea	Summer Tea	Autumn Tea		
1	1.613		Ethanethiol	MS	0.69 ± 0.06b	1.05 ± 0.05a	1.00 ± 0.04a	1.12	Sulfurous, fruity
2	1.678		Dimethyl sulfide	MS	19.38 ± 0.96b	22.10 ± 1.57a	5.91 ± 0.76a	0.99	Sulfurous, sweet corn
3	1.991		2−Methylfuran	MS	0.00 ± 0.00c	2.55 ± 0.05a	0.41 ± 0.23b	1.29	Chocolate
4	2.151		(2E,4Z)−Hexadiene	MS	1.48 ± 0.09b	1.43 ± 0.06b	4.61 ± 0.02a	0.95	
5	2.342		2−Methylbutanal	MS	2.02 ± 0.09b	3.14 ± 0.20a	1.27 ± 0.16c	1.16	Cocoa, coffee, nutty
6	2.632		1−Methyl−1−cyclohexene	MS	0.00 ± 0.00b	0.00 ± 0.00b	1.03 ± 0.11a	0.95	Citrus
7	2.65		2−Ethylfuran	MS	1.04 ± 0.22a	0.86 ± 0.75ab	0.00 ± 0.00b	0.72	Sweet, burnt, earthy, malty
8	3.651		3−Methyl−1−butanol	MS	0.00 ± 0.00c	1.99 ± 0.34a	1.02 ± 0.07b	1.25	Fruity
9	3.655		1−Pentanol	MS	2.53 ± 0.23a	1.98 ± 0.08b	0.88 ± 0.08c	0.91	Sweet, balsam
10	4.349		Methyl isobutenyl ketone	MS	7.02 ± 0.13a	6.68 ± 0.23a	2.51 ± 0.23b	0.94	Pungent, earthy
11	5.97	706	Leaf alcohol	MS, RI	1.49 ± 0.23a	0.95 ± 0.02b	1.31 ± 0.21a	1.08	Fresh, green, herbal, oily
12	6.408	721	Ethylbenzene	MS, RI	0.60 ± 0.04a	0.34 ± 0.01b	0.00 ± 0.00c	0.93	
13	6.625	729	2−Methyl−cyclopentanol	MS, RI	0.00 ± 0.00b	0.00 ± 0.00b	0.19 ± 0.10a	0.95	
14	7.939	768	Phenylethylene	MS, RI	1.06 ± 0.40a	0.83 ± 0.02a	0.26 ± 0.05b	0.80	Sweet, balsam, floral
15	8.064	771	Methoxy−phenyl−oxime	MS, RI	0.22 ± 0.01b	0.50 ± 0.02a	0.13 ± 0.01c	1.22	
16	8.155	773	Heptanal	MS, RI	1.82 ± 0.44a	0.65 ± 0.02b	0.31 ± 0.04b	0.99	Fresh, fatty, green, herbal
17	9.512	810	3,4−Dimethylphenol	MS, RI	0.00 ± 0.00b	0.00 ± 0.00b	0.75 ± 0.13a	0.94	Fatty
18	10.488	840	1,3−Dimethylbenzene	MS, RI	2.28 ± 0.30a	1.36 ± 0.27b	1.16 ± 0.10b	0.98	Plastic
19	11.238	862	Benzaldehyde	MS, RI	0.45 ± 0.01a	0.48 ± 0.03a	0.56 ± 0.45a	0.21	Sweet, almond, cherry
20	11.91	881	1−Octen−3−ol	MS, RI	1.12 ± 0.05a	0.12 ± 0.21c	0.48 ± 0.01b	1.20	Earthy, green, oily
21	12.218	889	Myrcene	MS, RI	4.92 ± 0.14a	4.92 ± 0.08a	4.88 ± 0.39a	0.09	Peppery, spicy, balsam
22	12.772	904	3,3,5−Trimethyl−1,5−heptadiene	MS, RI	0.63 ± 0.05b	0.81 ± 0.02a	0.56 ± 0.03c	1.17	
23	13.145	916	cis−Octahydropentalene	MS, RI	0.00 ± 0.00c	0.30 ± 0.01b	1.30 ± 0.02a	0.92	
24	14.026	943	o−Cymene	MS, RI	1.80 ± 0.20b	2.18 ± 0.17ab	2.60 ± 0.25a	0.83	
25	14.255	950	D−Limonene	MS, RI	4.30 ± 0.52b	5.83 ± 0.41a	4.99 ± 0.46ab	1.10	Citrus−like, fresh, sweet
26	14.611	960	Benzyl alcohol	MS, RI	0.48 ± 0.27a	0.28 ± 0.16a	0.25 ± 0.03a	0.60	Floral rose phenolic balsamic
27	14.68	962	(Z)−3,7−Dimethyl−1,3,6−octatriene	MS, RI	0.00 ± 0.00c	0.43 ± 0.16b	1.63 ± 0.80a	0.92	Floral, herb, sweet
28	15.192	976	4−Amino−2−methylphenol	MS, RI	4.26 ± 0.37a	0.00 ± 0.00b	0.00 ± 0.00b	1.12	
29	15.196	976	m−Anisidine	MS, RI	0.00 ± 0.00b	2.89 ± 0.27a	0.00 ± 0.00b	1.28	
30	16.05	999	2−Acetyl pyrrole	MS, RI	0.33 ± 0.66a	0.00 ± 0.00b	0.10 ± 0.07b	1.14	Licorice, walnut
31	16.527	1014	Cyclooctane	MS, RI	0.00 ± 0.00b	0.00 ± 0.00b	0.55 ± 0.01a	0.95	
32	16.544	1015	1−Octanol	MS, RI	0.00 ± 0.00b	0.54 ± 0.06a	0.00 ± 0.00b	1.26	Green
33	16.882	1025	3−Ethenyl−1,2−dimethyl−1,4−cyclohexadiene	MS, RI	0.00 ± 0.00b	0.00 ± 0.00b	0.47 ± 0.02a	0.95	
34	17.221	1035	Ethyl 2−(5−methyl−5−vinyltetrahydrofuran−2−yl) propan−2−yl carbonate	MS, RI	1.05 ± 0.04b	1.37 ± 0.20b	4.41 ± 0.39a	0.91	
35	17.433	1042	2,4−Dimethyl styrene	MS, RI	1.07 ± 0.28b	1.37 ± 0.17b	2.03 ± 0.05a	0.869	Spicy
36	18.075	1060	Linalool	MS, RI	4.42 ± 0.01a	4.28 ± 0.03a	3.56 ± 0.06b	0.91	Floral
37	18.113	1062	3,7−Dimethyl−1,5,7−octatrien−3−ol	MS, RI	1.30 ± 0.14b	2.05 ± 0.01b	14.53 ± 0.01a	0.94	
38	18.261	1066	Nonanal	MS, RI	3.01 ± 0.13a	2.19 ± 0.12b	0.00 ± 0.00c	0.91	Rose, fresh, orange, fatty
39	18.595	1075	2−Methyl−6−methylene−1,7−octadien−3−one	MS, RI	0.00 ± 0.00b	1.96 ± 0.13a	1.58 ± 0.81ab	0.93	
40	18.608	1075	Phenethyl alcohol	MS, RI	6.90 ± 0.19a	1.33 ± 0.01b	1.21 ± 0.04b	1.11	Floral, rose
41	18.981	1086	(3E,5E)−2,6−Dimethyl−1,3,5,7−octatetrene	MS, RI	0.56 ± 0.01b	0.98 ± 0.14b	5.31 ± 0.21a	0.93	
42	19.336	1095	(4E,6Z)−2,6−Dimethyl−2,4,6−octatriene	MS, RI	0.00 ± 0.00b	0.00 ± 0.00b	1.45 ± 0.58a	0.95	
43	19.778	1108	Benzyl cyanide	MS, RI	0.00 ± 0.00b	0.00 ± 0.00b	0.49 ± 0.03a	0.95	
44	20.221	1122	Methyl mandelate	MS, RI	0.00 ± 0.00b	0.00 ± 0.00b	0.36 ± 0.01a	0.95	
45	20.476	1130	Neroloxide	MS, RI	0.00 ± 0.00b	0.00 ± 0.00b	0.16 ± 0.03a	0.89	Green, herbal
46	21.608	1164	Linalool oxide	MS, RI	0.48 ± 0.02b	0.59 ± 0.05b	1.18 ± 0.02a	0.92	Floral, honey
47	21.608	1164	cis−5−Ethenyltetrahydro−α, α−5−trimethyl−2−furanmethanol	MS, RI	0.82 ± 0.13a	0.00 ± 0.00b	0.00 ± 0.00b	1.07	Earthy, floral, sweet, woody
48	21.738	1167	2,4,6−Trimethylstyrene	MS, RI	0.00 ± 0.00c	0.17 ± 0.00b	0.22 ± 0.02a	1.02	
49	22.189	1180	trans−3−Hexenyl butyrate	MS, RI	0.23 ± 0.06b	0.22 ± 0.02b	0.68 ± 0.03a	0.94	
50	22.566	1191	Methyl salicylate	MS, RI	0.62 ± 0.07b	0.89 ± 0.18b	2.49 ± 0.74a	0.85	Mint
51	22.574	1191	(−)−α−Terpineol	MS, RI	0.46 ± 0.01b	0.47 ± 0.01b	0.56 ± 0.01a	0.92	Floral
52	22.713	1195	2,3−Dihydro−2,2,6−trimethylbenzalhyde	MS, RI	0.42 ± 0.01a	0.36 ± 0.02b	0.00 ± 0.00c	0.93	Fresh, herbal, spicy
53	22.731	1195	2,4−Dimethyl−1−(1−methylethenyl)−cyclohexene	MS, RI	0.00 ± 0.00b	0.00 ± 0.00b	0.76 ± 0.01a	0.95	
54	22.908	1200	Dodecane	MS, RI	0.48 ± 0.38a	0.43 ± 0.01a	0.40 ± 0.02a	0.71	
55	23.199	1207	Decanal	MS, RI	0.22 ± 0.02b	0.23 ± 0.04ab	0.31 ± 0.05a	0.72	Sweet, orange, floral
56	23.286	1209	N−methoxycarbonyl−l−norleucine decyl ester	MS, RI	0.00 ± 0.00b	0.51 ± 0.01a	0.36 ± 0.02ab	1.22	
57	23.663	1217	β−Cyclocitral	MS, RI	0.47 ± 0.02a	0.31 ± 0.02ab	0.21 ± 0.17b	0.77	Herbal, clean, rose, sweet, fruity
58	23.754	1220	Methyl 2−methylvalerate	MS, RI	1.98 ± 0.03a	1.37 ± 0.11b	0.87 ± 0.06c	0.95	Fruity
59	24.135	1228	Terpinolene	MS, RI	0.40 ± 0.04b	0.29 ± 0.01c	1.14 ± 0.06a	0.98	
60	24.33	1232	cis−3−Hexenyl isovalerate	MS, RI	0.32 ± 0.04b	0.28 ± 0.03b	1.07 ± 0.20a	0.93	Fresh, green, apple fruity, pineapple
61	24.656	1240	3,6−Dimethoxy−9−(2−phenylethynyl)−fluoren−9−ol	MS, RI	0.00 ± 0.00c	0.22 ± 0.07b	0.66 ± 0.03a	0.91	
62	24.747	1242	5−Methylthiazole	MS, RI	0.29 ± 0.01a	0.00 ± 0.00c	0.16 ± 0.01b	1.28	
63	25.371	1255	Geraniol	MS, RI	0.43 ± 0.01a	0.00 ± 0.00c	0.22 ± 0.02b	1.27	Sweet, floral, fruity, rose, citrus
64	25.375	1255	2−Phenylethyl bromoacetate	MS, RI	0.35 ± 0.01a	0.00 ± 0.00ab	0.00 ± 0.00ab	1.12	
65	25.379	1255	2,6,6−Trimethyl−1−cyclohexene−1−acetaldehyde	MS, RI	0.00 ± 0.00b	0.00 ± 0.00b	0.16 ± 0.01a	0.95	Woody, fruity
66	26.246	1273	Nonanoic acid	MS, RI	0.35 ± 0.01a	0.00 ± 0.00b	0.00 ± 0.00b	1.12	
67	27.062	1290	Indole	MS, RI	0.71 ± 0.06b	1.77 ± 0.13a	0.75 ± 0.10b	1.27	Floral
68	27.33	1295	Theaspirane	MS, RI	0.28 ± 0.08ab	0.17 ± 0.01b	0.31 ± 0.05a	0.99	Herbal, green, woody, spicy
69	27.586	1300	Tridecane	MS, RI	0.66 ± 0.04a	0.69 ± 0.15a	0.00 ± 0.00b	0.94	
70	29.598	1347	Longipinene	MS, RI	0.26 ± 0.01a	0.22 ± 0.01b	0.20 ± 0.01b	0.91	
71	29.866	1353	α−Ionene	MS, RI	0.23 ± 0a	0.22 ± 0.01a	0.16 ± 0.01b	0.91	
72	30.456	1367	(+)−Cyclosativene	MS, RI	0.27 ± 0.01a	0.15 ± 0.01b	0.00 ± 0.00c	0.93	
73	30.469	1367	α−Ylangene	MS, RI	0.00 ± 0.00b	0.00 ± 0.00b	0.24 ± 0.02a	0.95	
74	30.642	1371	Longicyclene	MS, RI	0.39 ± 0.01a	0.30 ± 0.01b	0.26 ± 0.00c	0.99	
75	30.759	1373	α−Copaene	MS, RI	0.19 ± 0.01b	0.20 ± 0.01b	0.42 ± 0.04a	0.92	
76	31.119	1381	cis−3−Hexenyl hexanoate	MS, RI	0.81 ± 0.08b	0.87 ± 0.16b	1.52 ± 0.26a	0.85	Fruity, green, grassy
77	31.367	1386	Hexyl hexanoate	MS, RI	0.34 ± 0.04a	0.36 ± 0.03a	0.39 ± 0.03a	0.53	Herbal, fresh, grass, vegetable, fruity
78	31.557	1391	Jasmone	MS, RI	0.84 ± 0.07a	0.57 ± 0.03b	0.74 ± 0.08a	1.16	Woody, herbal, floral, spicy, jasmin
79	32.017	1400	Tetradecane	MS, RI	1.07 ± 0.08a	0.82 ± 0.06b	0.43 ± 0.03c	0.91	
80	32.156	1402	Longifolene	MS, RI	3.44 ± 0.15a	2.58 ± 0.28b	2.57 ± 0.10b	1.04	Sweet, woody, rose, medical
81	32.455	1407	α−Cedrene	MS, RI	0.40 ± 0.01a	0.33 ± 0.03b	0.30 ± 0.02b	0.96	Woody, cedar, sweet, fresh
82	32.606	1409	Caryophyllene	MS, RI	0.79 ± 0.04a	0.58 ± 0.09b	0.77 ± 0.04a	1.14	Sweet, woody, spice,
83	33.252	1419	2,6−Dimethyl−6−(4−methyl−3−pentenyl)−bicyclo [3.1.1]hept−2−ene	MS, RI	0.00 ± 0.00b	0.85 ± 0.15a	0.13 ± 0.10b	1.27	
84	33.955	1430	Geranylacetone	MS, RI	0.41 ± 0.01a	0.22 ± 0.02c	0.35 ± 0.04b	1.25	Fresh, green, fruity, rose, woody, magnolia
85	34.128	1433	Z,Z,Z−1,5,9,9−Tetramethyl−1,4,7,−cycloundecatriene	MS, RI	0.29 ± 0.02a	0.20 ± 0.00b	0.29 ± 0.03a	1.17	
86	35.143	1448	β−Ionone	MS, RI	0.26 ± 0.00a	0.18 ± 0.01c	0.22 ± 0.01b	1.24	Floral, woody
87	35.949	1459	α−Muurolene	MS, RI	0.00 ± 0.00b	0.00 ± 0.00b	0.21 ± 0.04a	0.94	
88	36.148	1462	Butylated hydroxytoluene	MS, RI	0.64 ± 0.001a	0.47 ± 0.03c	0.57 ± 0.05b	1.20	Camphor
89	36.357	1465	2,4−Di−tert−butylphenol	MS, RI	0.22 ± 0.01a	0.20 ± 0.01a	0.11 ± 0.02b	0.88	
90	36.716	1470	Isobutyl (m−tolyl) sulfide	MS, RI	0.47 ± 0.02a	0.00 ± 0.00b	0.00 ± 0.00b	1.12	
91	36.738	1470	d−Cadinene	MS, RI	0.00 ± 0.00c	0.26 ± 0.04b	0.60 ± 0.11a	0.91	Herbal, woody
92	36.864	1472	l−Calamenene	MS, RI	0.27 ± 0.03b	0.57 ± 0.09a	0.34 ± 0.06b	1.19	herb spice
93	37.445	1480	α−Murulene	MS, RI	0.00 ± 0.00b	0.00 ± 0.00b	0.15 ± 0.04a	0.92	
94	37.627	1482	α−Calacorene	MS, RI	0.00 ± 0.00b	0.00 ± 0.00b	0.14 ± 0.02a	0.94	Woody
95	40.12	1600	Hexadecane	MS, RI	0.23 ± 0.16a	0.14 ± 0.03a	0.16 ± 0.09a	0.48	

^1^ Retention indices as determined on DB−5MS column using the homologous series of n−alkanes (C_6_−C_25_). ^2^ Compounds are listed in order of retention time. ^3^ Method of identification: MS, mass spectrum comparison using NIST library; RI, retention index in agreement with NIST library. ^4^ Relative contents of the identified compounds were obtained by dividing the area of a single peak by the total areas. The content of volatile compounds are represented as the mean value ± standard deviation (mean ± SD). Different small letters indicate significant differences (*p* < 0.05). ^5^ Odor description data from http://cosylab.iiitd.edu.in/flavordb (accessed on 20 November 2021).

## Data Availability

The data supporting the results of this study are included in the present article.
